# Autologous Tolerogenic Dendritic Cells for Rheumatoid Arthritis-2 (AuToDeCRA-2) study: protocol for a single-centre, experimental medicine study investigating the route of delivery and potential efficacy of autologous tolerogenic dendritic cell (TolDC) therapy for rheumatoid arthritis

**DOI:** 10.1186/s13063-025-08972-x

**Published:** 2025-08-07

**Authors:** Ema-Louise Long, James Stanway, Michael White, Nicola Goudie, Julia Phillipson, Miranda Morton, Asma Abdul Malek, Philip Brown, Geoff Hide, Ralph Jackson, Colin Nice, John Tuckett, Julie Diboll, Amy Anderson, Arthur Pratt, Catharien M. U. Hilkens, John Isaacs

**Affiliations:** 1 Translational & Clinical Research Institute, Faculty of Medical Sciences, Newcastle University, Newcastle Upon Tyne, UK; 2 Department of Rheumatology, Freeman Hospital, Newcastle Upon Tyne, UK; 3 Newcastle Clinical Trials Unit, Newcastle University, Newcastle Upon Tyne, UK; 4 Clinical Research Facility, Royal Victoria Infirmary, Newcastle Upon Tyne, UK; 5 Department of Radiology, Newcastle Upon Tyne Hospitals NHS Foundation Trust, Newcastle Upon Tyne, UK; 6 NIHR Newcastle Biomedical Research Centre, Newcastle Upon Tyne, UK

**Keywords:** Rheumatoid arthritis (RA),, Tolerogenic dendritic cells (TolDC),, Shared epitope (SE),, Anti-Citrullinated Peptide Antibody (ACPA),, Immunomodulation,, Leukapheresis,, Intra-nodal,, Intra-articular,, Intra-dermal,, Lymph node

## Abstract

**Background:**

Dendritic cells are professional antigen presenting cells with the ability, in their immature state, to induce tolerance in T cells. A protocol to develop phenotypically stable tolerogenic dendritic cells (TolDC) was developed in Newcastle and cells administered to participants in the phase I AuToDeCRA study, demonstrating that TolDC were safe and well tolerated. More knowledge of the TolDC product is now needed, such as optimal dose, route of administration and antigen loading. Establishing this and developing a biomarker profile to demonstrate favourable immunomodulation is the focus of AuToDeCRA-2.

**Methods:**

AuToDeCRA-2 is a non-commercial, phase IIa, 5-arm, randomised, unblinded, single-centre study. It is designed to demonstrate and compare immunomodulation achieved by TolDC administered via three distinct routes: intra-nodal, intra-articular, intra-dermal and, in the case of intra-nodal administration, at 2 doses. Participants will be randomised to one of these four active intervention arms or standard care. Participants in intervention arms will receive a single dose of TolDC loaded with synthetic citrullinated peptides (TolDC_CitPep_) representing disease relevant autoantigens. Twenty Anti-Citrullinated Peptide Antibody (ACPA) positive, shared epitope positive Rheumatoid Arthritis patients with nil-to-moderate disease activity will be randomised in an allocation ratio of 1:1:1:1:1.

Participants will be followed up with immune state monitoring performed on peripheral blood samples at baseline, 1, 3 and 6 weeks and lymph node aspirates at baseline and 1 week, alongside clinical assessment performed throughout and additionally at 12 weeks.

**Discussion:**

TolDC therapy is an emerging cellular therapy aimed at reversing the underlying abnormality in autoimmune disease by inducing tolerance to autoantigen. Expected challenges to this study include recruitment of potentially asymptomatic participants to a complex and intensive experimental medicine study. Limitations include the relatively small number of participants although adequate to address the aims of the study. Establishing acceptable route(s) of administration as well as demonstrating favourable immunomodulation via the development of a biomarker profile is the focus of AuToDeCRA-2, which aims to address some of the existing scientific gaps necessary for the development of TolDC therapy in autoimmune disease.

**Trial registration:**

ISRCTN, ISRCTN14999554. Registered on 27th September 2023.

## Administrative information

Note: the numbers in curly brackets in this protocol refer to SPIRIT checklist item numbers. The order of the items has been modified to group similar items (see http://www.equator-network.org/reporting-guidelines/spirit-2013-statement-defining-standard-protocol-items-for-clinical-trials/).

**Table:** 

Title {1}	A single-centre, experimental medicine study investigating the route of delivery and potential efficacy of autologous tolerogenic dendritic cell (TolDC) therapy for Rheumatoid Arthritis-2 (AuToDeCRA-2)
Trial registration {2a and 2b}.	ISRCTN14999554. Registered on 27th September 2023. https://doi.org/10.1186/ISRCTN14999554
Protocol version {3}	V5.0 31 st July 2024
Funding {4}	Versus Arthritis (formally Arthritis Research UK) ref: 21811 RTCure (Rheuma Tolerance for Cure) as part of the Innovative Medicines Initiative 2 Joint Undertaking ref:777357
Author details {5a}	^1^Translational & Clinical Research Institute, Faculty of Medical Sciences, Newcastle University, United Kingdom ^2^Department of Rheumatology, Freeman Hospital, Newcastle upon Tyne, United Kingdom ^3^Newcastle Clinical Trials Unit, Newcastle University, United Kingdom ^4^Clinical Research Facility, Royal Victoria infirmary, Newcastle upon Tyne, United Kingdom ^5^Department of Radiology, Newcastle upon Tyne Hospitals NHS Foundation Trust, Newcastle upon Tyne, United Kingdom ^6^NIHR Newcastle Biomedical Research Centre, Newcastle upon Tyne, United Kingdom
Name and contact information for the trial sponsor {5b}	The Newcastle upon Tyne Hospitals NHS Foundation Trust Sponsor reference: 08669 tnu-tr.sponsormanagement@nhs.net
Role of sponsor {5c}	The sponsor has no role in the study design; collection, management, analysis and interpretation of data; writing of the report or the decision to submit for publication

## Introduction

### Background and rationale {6a}

Autoimmune disease such as rheumatoid arthritis (RA) results from a breakdown of immunological tolerance; the immune system mistakenly recognises healthy, self-tissue as a threat, leading to inflammation and eventually structural damage. No current treatment option for RA represents a cure and medication is required indefinitely, carrying with it the risks of generalised immunosuppression. It is desirable to develop ‘tolerogenic’ treatments, which reverse the underlying abnormality of the immune system rather than continuous non-specific suppression of inflammation and immunity. Tolerogenic dendritic cells (TolDC) have emerged as one such possibility [1–7].

CD4^+^ helper T cells are a key driver of disease in RA, in keeping with their established role as orchestrators of adaptive immune responses through their interaction with professional antigen presenting cells (APC). As key APC, dendritic cells (DC) instruct CD4^+^ T cells how to respond to a given antigen by modulating the co-stimulation they provide. Depending on context, DCs can induce either proinflammatory or regulatory (tolerogenic) characteristics in the T cells [8]. If an antigen is encountered in the context of threat, the DC in question ‘matures’ and will present antigen with co-stimulatory signals, which leads to the generation of antigen-specific T cells with pro-inflammatory characteristics. Conversely, if the antigen is encountered in a non-threatening microenvironment, the DC remains ‘immature’ and upon interaction with the T cell, may induce a regulatory phenotype, anergy (a state of hyporesponsiveness) or even apoptosis. With their ability to influence the adaptive immune system in an antigen-specific way, DCs are an exciting potential therapeutic tool which could ‘reset’ autoimmunity.

Whilst immature DCs can induce T cell tolerance, their phenotype is unstable, and they may develop into immunogenic, mature DCs in the presence of pro-inflammatory cytokines. As such, a variety of protocols have been developed for the generation of phenotypically stable TolDC which retain their tolerogenic characteristics, even in a pro-inflammatory microenvironment. We have established such a protocol [9–12] and these cells were administered safely to patients with inflammatory arthritis in the earlier phase I AuToDeCRA study [1].

In AuToDeCRA, TolDC were loaded with autologous synovial fluid as a source of antigen and administered arthroscopically into an inflamed knee joint following saline wash-out. The lack of a subsequent disease flare following administration suggested that, as shown by in vitro studies, the TolDC phenotype was stable. These findings have been corroborated by several other international studies using TolDC in patients with RA and other autoimmune diseases such as multiple sclerosis and type 1 diabetes [3–7, 13]. Although no TolDC trial has yet demonstrated robust clinical efficacy, TolDC appear to be a safe and well tolerated intervention.

There is now a need to demonstrate the ability of TolDC to achieve immunomodulation. Tolerogenic therapies are not necessarily anti-inflammatory in the short term [14] and biomarkers of the immune state that are influenced more rapidly by such treatments are urgently needed, not least to guide clinical protocol development relating to aspects such as optimal dose, route of administration and antigen loading. AuToDeCRA-2 was developed to begin to address some of these questions.

### Objectives {7}

AuToDeCRA-2 is designed to demonstrate and compare immunomodulation using synthetic citrullinated peptide-loaded TolDC (TolDC_CitPep_) administered via three distinct routes: intra-nodal, intra-articular, intra-dermal and in the case of intra-nodal administration, at a low and a high dose. The rationale for loading TolDC with citrullinated peptides is explained in section ‘Intervention description {11a}’.

Table 1 outlines the study objectives which will be achieved by comparing baseline data with follow-up data at 1, 3, 6 and 12 weeks.

**Table 1:** Study objectives

Primary objectives	Secondary objectives	Exploratory objectives
• To seek signals of immune modulation when TolDC_CitPep_ are administered to participants with RA using immune state biomarkers performed on peripheral blood samples at baseline, 1, 3 and 6 week follow-up and participant lymph node aspirates at baseline and 1 week follow-up	• To seek signs of clinical efficacy when TolDC_CitPep_ are administered to participants with RA using ACR 20, 50 and 70, DAS-28, and components thereof at baseline and 1, 3, 6 and 12 week follow-up • To provide further evidence of TolDC_CitPep_ safety using reported adverse events (AEs) at baseline, 1, 3, 6, and 12 week follow-up • To provide further evidence of participant acceptability of TolDC_CitPep_ therapy for RA using a Participant Acceptability questionnaire at 12 week follow-up	• To link signals of immune modulation with signs of potential efficacy of TolDC_CitPep_ using potential associations between immune modulation measures and evidence of efficacy for the three different routes of TolDC_CitPep_ administration (and, in the case of intra-nodal injection, for the two doses administered) • To compare intra-nodal (low and high dose), intra-dermal and intra-articular injection with regards to primary and secondary objectives, and feasibility

### Trial design {8}

AuToDeCRA-2 is a non-commercial, exploratory, unblinded, randomised experimental medicine study with 5 parallel groups of ACPA-positive RA participants in an allocation ratio of 1:1:1:1:1, with the aim of demonstrating and comparing immunomodulation by TolDC_CitPep_.

The study protocol follows the Standard Protocol Items: Recommendations for Interventional Trials (SPIRIT) 2013 statement. The study has been registered with ISRCTN [14999554].

## Methods: participants, interventions and outcomes

### Study setting {9}

The single-centre study will be undertaken at Newcastle upon Tyne Hospitals NHS Foundation Trust (NuTH) in the UK. All study visits will take place within a designated Clinical Research Facility at the Royal Victoria Infirmary, Newcastle upon Tyne, with the exception of leukapheresis visits which will take place in the Leukapheresis Day Unit at the Freeman Hospital, Newcastle upon Tyne.

### Eligibility criteria {10}

#### Inclusion criteria

Patients are eligible for the study if all of the following apply at screening:

1. Adults aged 18 years old or over
2. RA fulfilling 1987 ARA criteria or 2010 ACR/EULAR Classification Criteria
3. ACPA > 3 × upper limit of normal, can include historical measurements providing the result remains positive at screening
4. Able and willing to give informed consent and to comply with the study protocol
5. Disease duration of at least 4 months and less than 10 years
6. ACR Functional Class I-III
7. DAS-28 < 5.1
8. If receiving Disease Modifying Anti-Rheumatic Drugs, these can include any combination of methotrexate, sulphasalazine, azathioprine, hydroxychloroquine, abatacept, rituximab (last dose > 6 months ago), TNF-alpha inhibitors and IL6 receptor antagonists, provided stable dosing for at least 4 weeks
9. Possess at least one copy of a shared epitope (SE) HLA DRB1 allele (0101; 0102; 0105; 0401; 0404; 0405; 0408; 0409; 0410; 0413; 0416; 0419; 0421; 1001; 1402; 1406; 1409; 1413; 1417; 1419; 1420; 1421)

#### Exclusion criteria

Patients are excluded from the study if any of the following apply at screening:

1. Use of other investigational medicinal products within 30 days prior to study entry (defined as date of consent into study)
2. Any changes to RA treatment within 4 weeks of study entry
3. Current treatment with Janus kinase inhibitors or leflunomide. Previous treatment is permitted provided at least 12 weeks have elapsed at study entry since discontinuation
4. Receiving glucocorticoids by any route within 4 weeks of study entry, apart from topical, intra-nasal or inhaled
5. Serious or unstable co-morbidity that prohibits participation in the study at the discretion of the investigator, e.g. significant chronic obstructive pulmonary disease, significant cardiac failure, active malignancy
6. Active infection at study entry (except fungal nail infection)
7. Infection requiring hospitalisation or intravenous antibiotics within 4 weeks prior to study entry
8. Immunisation with live, attenuated vaccines planned within 14 days of baseline visit (administration of TolDC_CitPep_) and with non-live vaccines planned within 7 days of baseline visit
9. History of hepatitis B or C, HIV or HTLV-1/2 infection(s)
10. Recent history of cytomegalovirus (CMV) infection (positive for CMV IgM antibodies) or syphilis infection (positive PCR test)
11. Major surgery within 8 weeks prior to study entry or planned within 12 weeks of baseline visit
12. Pregnancy, or women planning to become pregnant within the study period, or women who are breast feeding
13. Females of childbearing potential engaging in heterosexual relationships unwilling to use adequate contraception for the duration of the study
14. Patients taking anticoagulants that cannot be interrupted and are, in the judgement of the investigator, likely to interfere with study procedures
15. Known hypersensitivity to local anaesthetic
16. Poor venous access or medical condition precluding leukapheresis, e.g. unstable cardiac arrythmia (atrial fibrillation permitted)
17. Haemoglobin < 10 g/dL; neutrophils < 1.00 × 10^9^/L; platelets < 100 × 10^9^/L

Radiologists performing ultrasound-guided lymph node aspiration and intra-nodal tolDC administration will be competent in the procedure.

Rheumatologists performing intra-articular administration will be competent at performing ultrasound guided intra-articular (knee) injection.

Clinicians performing intra-dermal administration will be competent at intra-dermal injections and have received training on the use of the specific intra-dermal needle selected for use.

### Who will take informed consent? {26a}

Following initial contact, and at least 24 h after provision of participant information documents, interested participants will attend a screening visit. At the screening visit, a member of the study team will ensure the participant information sheet (PIS) has been read and understood and answer any questions. If the participant wishes to enter the study, written informed consent will be obtained by a medically qualified member of the study team delegated to the task.

### Additional consent provisions for collection and use of participant data and biological specimens {26b}

There are 4 optional consent clauses which form part of the informed consent form (ICF) but do not affect eligibility to participate in the study.

1. Agreeing to contact by the study team about participation in additional follow-up visits in the future, should additional research funding be obtained for these visits.
2. Agreeing that any research samples remaining at the end of the study can be anonymised and sent to a biobank for long-term storage and use in future research outside of the study.
3. Agreeing that any samples left over from TolDC manufacture can be anonymised and sent to a biobank for long-term storage and use in future research outside of the study.
4. Agreeing to be sent a summary of the results when the study has finished.

## Interventions

### Explanation for the choice of comparators {6b}

The optimal route for TolDC administration is not clear. The DC-T-cell interaction is believed to occur primarily in lymph nodes. We hypothesise that the route of injection most likely to ensure the TolDC reach a lymph node is direct injection into lymph nodes, but intra-nodal injection is a less convenient route of delivery, requiring ultrasound equipment and a trained operator. AuToDeCRA-2 will therefore compare direct intra-nodal injection with alternative routes that should enable cells to reach disease-relevant lymph nodes. Intra-dermal is the most well studied route of administration [5, 6, 15] for therapeutic DC and does not require specialised equipment. In contrast, intra-articular injection requires specialised equipment and operators, but it is possible that the joint-relevant immune system is best accessed via this route. 10^7^ TolDC will be administered via all routes to provide a direct comparison, being the highest dose that was administered in AuToDeCRA. A lower dose of 10^5^ TolDC will also be administered intra-nodally as injecting directly into a lymph node may require fewer cells to achieve a therapeutic effect.

The fifth arm is standard care. Standard care was chosen over placebo so that participants would not need to undergo unnecessary leukapheresis or administration procedures. Standard care involves no leukapheresis visit and no administration of TolDC_CitPep_ but all other study procedures remain the same.

### Intervention description {11a}

Eligible participants will be randomised in equal proportions to one of four intervention arms or standard care. Participants’ usual medication will be continued alongside for all groups.

Participants randomised to an intervention arm will attend for leukapheresis at day − 8 to extract CD14^+^ monocytes for the generation of TolDC_CitPep_. Leukapheresis products will be transferred to Newcastle Advanced Therapies Good Manufacturing Practice (GMP) Facility where the TolDC_CitPep_ will be manufactured.

In AuToDeCRA and AuToDeCRA-2, TolDC manufacture is identical aside from the antigen loaded into TolDC. In AuToDeCRA-2, the TolDC will be loaded with synthetic citrullinated peptides (TolDC_CitPep_) representing disease-relevant epitopes [16–19] rather than autologous synovial fluid as in AuToDeCRA. Knowledge of the loaded antigen enables more sophisticated immune monitoring assays that incorporate measures of antigen specificity, allowing the profiling of the cells the treatment is designed to target. Furthermore, treatment is no longer limited to patients with a knee joint effusion, enabling treatment of participants with lower disease activity that may benefit most from tolerance inducing strategies. In vitro validation work has confirmed the immunological equivalence of AuToDeCRA and AutoDeCRA-2 TolDC.

TolDC_CitPep_ loaded with Cit-alpha-enolase (326–340), Cit-cartilage intermediate layer protein-2 (297–311), Cit-vimentin (59–78) and Cit-tenascin-C 22 (1012–1026) will be diluted to a final volume of 200 µl, aspirated into a syringe and undergo quality control and QP release. The syringe will be stored at 2–8 °C in a validated and monitored refrigerator until administration. Administration by all injection routes will be carried out immediately after (inguinal) lymph node aspiration (for biomarker analysis), following which participants will be observed for at least 90 min.

- Intra-nodal injection of TolDC_CitPep_ (10^5^ or 10^7^ cells).
  TolDC_CitPep_ will be injected into up to 5 inguinal lymph nodes under ultrasound visualisation, on the ipsilateral side to the lymph node aspiration as per intra-nodal aspiration and injection Standard Operating Procedure (SOP).
- Intra-articular injection of TolDC_CitPep_ (10^7^ cells).
  TolDC_CitPep_ will be administered as a single injection into a knee joint under ultrasound visualisation as per intra-articular injection SOP. The knee selected should be the least inflamed, and ipsilateral to the aspirated lymph node where possible.
- Intra-dermal injection of TolDC_CitPep_ (10^7^ cells).
  Intra-dermal injection will be administered as one or two injections to the upper, anterior thigh (within 5 cm of the inguinal crease) on the ipsilateral side as the lymph node aspiration using an intra-dermal 1.2-mm silicon needle as per intra-dermal injection SOP.

### Criteria for discontinuing or modifying allocated interventions {11b}

There are no plans to modify or permit modification of allocated interventions. The intervention will not be administered if the TolDC_CitPep_ do not meet stringent release criteria.

### Strategies to improve adherence to interventions {11c}

The intervention is administered as a single dose in a clinical research facility. It is felt unlikely that a participant would not attend for administration having undergone leukapheresis.

### Relevant concomitant care permitted or prohibited during the trial {11d}

Any medication or concomitant care not specifically listed is presumed to be permitted.

Permitted concomitant care:

- Permitted treatments for RA include methotrexate, sulfasalazine, azathioprine, hydroxychloroquine, abatacept, rituximab (last dose > 6 months ago), TNF-alpha inhibitors, and IL6 receptor antagonists, alone or in combination, at a stable dose for at least 4 weeks
- Anticoagulants unless these are, in the view of the investigator, likely to interfere with study procedures and cannot be interrupted

Prohibited concomitant care:

- Glucocorticoids (apart from topical, intra-nasal, inhaled)
- Janus kinase inhibitors
- Leflunomide
- Live, attenuated vaccines within 2 weeks of and non-live vaccines within 1 week of the baseline visit

### Provisions for post-trial care {30}

Any unexpected or abnormal test results will be discussed with the participant and any necessary follow-up arranged with their rheumatologist, GP or another specialist. Participants will continue to access their usual health practitioners in the normal way throughout and after the study.

No additional TolDC product will be made available post-trial.

NHS indemnity for clinical trials will apply for clinical negligence that harms individuals towards whom the NHS has a duty of care.

### Outcomes {12}

#### Primary Endpoint/Outcome

There is currently no agreed biomarker for tolerance induction. Pragmatically, we shall seek evidence of favourable immunomodulation by selecting from a variety of different modalities including:

- Increase in proportional abundance of regulatory T cells in peripheral blood by high dimensional cytometry
- Induction or increase in Interleukin-10 production in peripheral blood mononuclear cells (PBMCs) following citrullinated peptide stimulation, using ELISpot or intracellular flow cytometry
- Reduction in interferon gamma production in PBMCs following citrullinated peptide stimulation
- Change in proliferative response of PBMCs to citrullinated peptide stimulation, combined with phenotypic (surface marker, cytokine production) correlation
- A reduction in ACPA titre or diversity of ACPA specificities
- Changes in circulating cytokine levels

#### Secondary Endpoints/Outcomes

Secondary outcome measures for the study are:

- Change in ACR 20, 50 and 70, DAS-28, and components thereof at weeks 1, 3, 6 and 12 compared to baseline
- Number of reported AEs. AEs will be collected at each timepoint (baseline, 1, 3, 6 and 12 weeks) from clinical assessment and routine blood testing
- Participant reported acceptability of the TolDC_CitPep_ product and study-related procedures including leukapheresis and mode of administration. Data will be collected at the 12-week visit via the Participant Acceptability questionnaire

#### Exploratory Endpoint/Outcome Measures

Exploratory outcome measures for the study are:

- Enumeration and phenotypic analysis of autoreactive T cells by major histocompatibility complex class II tetramer staining
- Single cell RNA sequencing of peripheral blood T cells and lymph node aspirates
- Potential associations between immune modulation (as defined by primary outcome measures) and evidence of efficacy (as defined by secondary outcome measures) for (1) the different routes of TolDC_CitPep_ administration (intra-articular, intra-dermal and intra-nodal) and (2) the different doses of TolDC_CitPep_ administered via the intra-nodal route (10^5^ and 10^7^)
- Comparison of (1) the different routes of TolDC_CitPep_ administration (intra-articular, intra-dermal and intra-nodal) and (2) the different doses of TolDC_CitPep_ administered via the intra-nodal route (10^5^ and 10^7^), for evidence of immune modulation and potential efficacy

### Participant timeline {13}

**Fig. 1:**
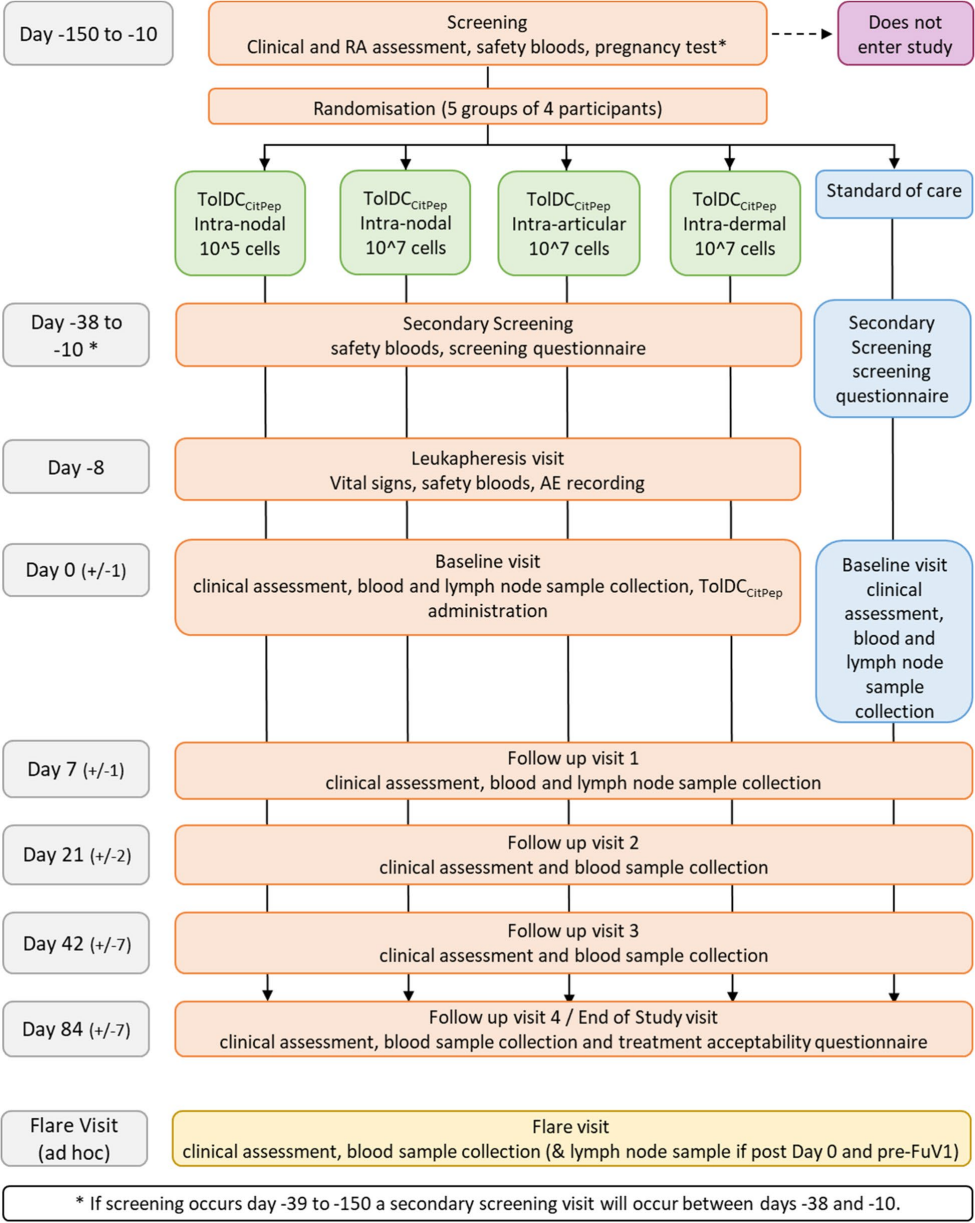
Trial flowchart

**Fig. 2:**
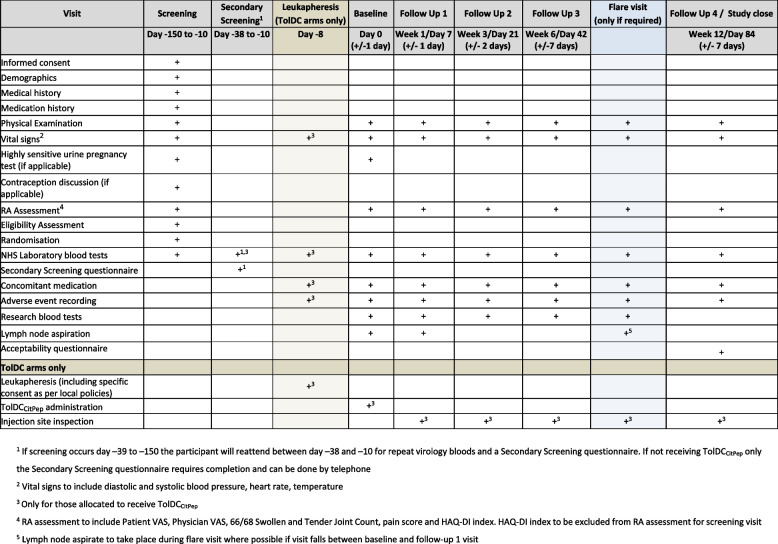
Schedule of events

The study flowchart (Fig. 1) outlines the participant journey through the study and the schedule of events (Fig. 2) outlines the activities at each study visit.

Screening will take place between day − 150 and day − 10. If screening takes place between day − 150 and day − 39 then a secondary screening visit will occur between day − 38 and day − 10. This is to ensure that the participant remains eligible and to allow for safety blood sampling in line with leukapheresis and TolDC production facility SOPs.

After screening and randomisation (and leukapheresis for those receiving TolDC_CitPep_), participants will attend for baseline (day 0) clinical and immunological assessments. All participants will provide a blood sample and an ultrasound-guided fine needle aspirate from inguinal lymph nodes. These blood and lymph node aspirate samples will contribute to the immunological endpoints described above. Participants in intervention arms will then receive TolDC_CitPep_ at the allocated dose and via the allocated administration route.

Participants will be followed up on four occasions at 1, 3, 6 and 12 weeks after the baseline visit. Clinical assessment and blood sampling will take place at each visit. At the week 1 follow-up visit, all participants will undergo a second lymph node ultrasound-guided fine needle aspirate.

If, during the follow-up period, a participant suffers deterioration in their arthritis symptoms, they will attend for a ‘flare visit’. A full clinical assessment will be performed and, if a flare of RA is diagnosed, the participant managed appropriately at the discretion of the investigator, according to standard care. If therapeutic intervention is required for the flare, final samples will be taken for immunological endpoints and the participant will exit the study.

### Sample size {14}

There are no relevant background data available to power this study, which is exploratory and not powered for statistical significance. The sample size of 20 is based upon expected feasible recruitment into a complex experimental medicine protocol, based on prior experience from AuToDeCRA.

### Recruitment {15}

A number of strategies will be implemented to identify potential participants:

1. Identification by usual Rheumatology care team
2. Identification via local Rheumatology departmental databases followed by a mailed study invitation letter from their usual care team and PIS. The letter will include a tear off slip and a stamped return envelope to express a wish for no further contact if applicable
3. Identification via National Institute for Health Research immune-mediated inflammatory diseases BioResource with consent for contact passed on by BioResource team
4. Ethically-approved advertisement via posters and leaflets including study contact details displayed in the Rheumatology out-patient department and via collaboration with relevant charities, patient groups and Clinical Research networks
5. Participant Identification Centre sites may be utilised to maximise recruitment via the Northern Regional Rheumatology Network, a group of rheumatology centres within the North East of England

## Assignment of interventions: allocation

### Sequence generation {16a}

Eligible participants will be randomly allocated to one of five groups. Randomisation will be computer generated and performed by delegated and trained members of the research team using a 24-h, central, secure, web-based system (Sealed Envelope™).

- Group 1 will receive 10^5^ TolDC_CitPep_ administered into inguinal lymph node/s
- Group 2 will receive 10^7^ TolDC_CitPep_ administered into inguinal lymph node/s
- Group 3 will receive 10^7^ TolDC_CitPep_ administered intra-articularly into a knee
- Group 4 will receive 10^7^ TolDC_CitPep_ administered intra-dermally into the thigh
- Group 5 will receive standard care and no active intervention (control group)

### Concealment mechanism {16b}

Allocation concealment will be ensured by the use of a centralised web-based service for randomisation (Sealed Envelope™).

### Implementation {16c}

Participants will be enrolled and randomised by delegated and trained members of the research team. The allocation sequence will be generated by Sealed Envelope™.

## Assignment of interventions: blinding

### Who will be blinded {17a}

The study is not blinded. Due to the need for those receiving TolDC_CitPep_ to undergo leukapheresis and TolDC_CitPep_ administration procedures via distinct routes, it is not practical to perform a blinded study.

### Procedure for unblinding if needed {17b}

The study is unblinded throughout.

## Data collection and management

### Plans for assessment and collection of outcomes {18a}

All assessments will be performed by a qualified healthcare professional delegated to the task.

Demographics, medical history and medication history will be collected at screening.

Clinical assessment will occur at screening, baseline, week 1, 3, 6 and 12 and any flare visit as required, and include discussion of any new or relevant clinical history including medications, physical examination, RA assessment and vital signs. Physical examination may vary in detail depending upon individual clinical history and will be selected to ensure a comprehensive understanding of participant health.

Assessment of the participant’s RA condition will include the following:

- Participant global assessment of disease activity visual analogue scale (VAS)—score 0–100 mm
- Physician global assessment of disease activity VAS—score 0–100 mm
- Pain VAS—score 0–100 mm
- 66/68 Swollen and Tender Joint Count
- Health Assessment Questionnaire Disability Index (HAQ-DI) (not required at screening)—score 0–3 rounded to one decimal place

Participant reported acceptability will take place at week 12 via a study-specific participant completed Acceptability Questionnaire consisting of sequential questions with a score to denote acceptability and a free text box where participants are invited to comment on any aspect of their involvement.

Full details of blood and lymph node aspirate sample collection are provided in section ‘Plans for collection, laboratory evaluation and storage of biological specimens for genetic or molecular analysis in this trial/future use {33}’.

The described assessments alongside the biological specimens will enable the collection of outcomes as set out in section ‘Outcomes {12}’.

### Plans to promote participant retention and complete follow-up {18b}

The clinical research team will discuss appointment dates with participants to ensure suitability, provide appointment dates in advance and address any transport or accessibility needs. Dates and times can be provided in writing. Reminder phone calls will be made prior to leukapheresis and baseline appointments. Future appointment dates and times will be re-discussed at each appointment.

Participants can withdraw from the study at any time without providing a specific reason. Data collected to the point of withdrawal will be retained and utilised, although participants may request to have stored samples destroyed. Participants withdrawing will be asked for written permission to collect and use data from routine clinical follow-up for the period of their intended participation. Where the TolDC_CitPep_ have been administered, contact in line with a participant’s week 12 assessment will be attempted to check for adverse reactions (ARs).

### Data management {19}

All data for an individual participant will be recorded in the study-specific electronic Case Report Form (eCRF) set up using Sealed Envelope’s™ Red Pill system which will include tested range checks for numerical data values. Access to the study database will be password-limited, with task-specific restrictions. Only staff formally delegated to do so will have access to the database.

Data will be handled, computerised and stored in accordance with the UK Data Protection Act 2018, UK GDPR, the latest GCP Directive (2005/28/EC) and local site policy. Paper copies of study-related documentation will be annotated, signed, dated and filed in the Investigator Site File (ISF). The signed ICF, eligibility forms, GP letter and RA assessments will be uploaded to the participant’s medical notes.

All study documentation will be archived for 30 years in accordance with UK GCP legislation and local SOPs.

### Confidentiality {27}

Participant identification on the eCRF will be via a unique study identifier number. A record linking the participant’s name to the unique study identifier number will be held in a locked room at the study site. Only the Principal Investigator (PI) and appropriately delegated staff will have access to source data and the ISF for the purpose of conducting the study.

### Plans for collection, laboratory evaluation and storage of biological specimens for genetic or molecular analysis in this trial/future use {33}

- NHS laboratory and tissue typing bloods

Participants will provide NHS laboratory blood samples, which will be analysed in local Trust laboratories and then discarded. These will be collected and tested as per Table 2 below.

**Table 2:** NHS laboratory blood samples

Blood set	Tests to be performed	Time points collected	Participants
Set 1	• Cyclic Citrullinated Peptide Antibody (Anti-CCP) • Rheumatoid factor (RhF) • Full blood count (FBC) • Erythrocyte sedimentation rate (ESR) • Coagulation Screen (with derived Fibrinogen) • International normalised ratio (INR) • Urea and electrolytes (U + E) • Magnesium (Mg) • Adjusted calcium • Liver function test (LFT) • C-reactive protein (CRP) • Human immunodeficiency virus (HIV) • Hepatitis B + C • Human T cell Leukaemia Virus (HTLV) 1 + 2 • Syphilis • Cytomegalovirus (CMV) • HLA DRB1 tissue type (except where tissue type is already known) • Follicle-stimulating hormone (FSH) *	Screening	All * FSH can be considered to prove post menopausal state in women < 50 years who have been amenorrhoeic for 12 months and would prefer testing to using contraception for the duration of the study on discussion with the investigator
Set 1B	• HIV • Hepatitis B + C • HTLV 1 + 2 • Syphilis • CMV	Secondary screening visit	Those screened > 38 days before scheduled baseline visit
Set 2	• FBC • U&E • LFT • Phosphate • Mg • Adjusted calcium • Group + Save • HIV • Hepatitis B + C • HTLV 1 + 2 • Syphilis	Leukapheresis visit	All, except participants randomised to Group 5 (control group)
Set 3	• FBC • U&E • LFT • CRP • ESR	Baseline, follow-up visits 1, 2 and 3	All
Set 4	• FBC • U&E • LFT • CRP • ESR • RhF • ACPA	Follow-up visit 4	All
Set 5	• FBC • U&E • LFT • CRP • ESR • Any additional test at the discretion of the investigator	Flare visit	Those completing a flare visit

- Peripheral blood research biomarkers

Research blood samples will be obtained at baseline and weeks 1, 3 and 6. This will involve the collection at each visit of 108 ml of blood in a combination of EDTA and serum separator tubes. Some of this sample will be studied immediately within the research laboratory and the remainder processed and cryopreserved for analysis following study completion.

- Lymph node aspirate samples

Lymph node aspirate samples will be obtained by an experienced radiologist using direct ultrasound visualisation to aspirate up to 5 lymph nodes using a 23 Gauge needle and 3-ml syringe under tension. Samples will be immediately passed to a member of the study team for processing and cryopreservation for analysis following study completion.

## Statistical methods

### Statistical methods for primary and secondary outcomes {20a}

AuToDeCRA-2 is not powered for formal statistical significance. Nonetheless, a statistical analysis plan will be developed during the study.

The analysis population will include all subjects who have received their allocated intervention. For AE reporting, the analysis population will be all individuals who provide consent, whether or not they subsequently receive TolDC_CitPep_.

Demographic characteristics, medical history and clinical characteristics collected at screening will be summarised descriptively and tabulated. For categorical variables, the frequency and percentage in each group will be reported. For continuous variables the mean, standard deviation and/or median and interquartile range will be reported.

Within each arm (*n* = 4), we will assess whether there are any consistent trends in the parameters being measured, both for primary and secondary outcomes, and we will compare each of the 4 intervention arms against the control group and each other for primary and secondary outcomes.

Summary statistics capturing differences in immune biomarkers (expressed as percentages, stimulation indices, titres, etc.) and clinical outcomes (descriptive and numeric) will be tabulated and presented graphically. Longitudinal analysis will be performed to compare samples following TolDC_CitPep_ administration at baseline. Given the low participant number in each group (4), no formal statistical testing will be performed other than to explore analysis methods, identify prospective transformations of the data, identify key prospective time points and obtain estimates of the variability in both primary and secondary measures to inform the design of a future efficacy study. Estimates of variability will be based on a mixed effects model including participant, route of administration and sampling time point.

### Interim analyses {21b}

There is no planned formal interim analysis.

Participant flow through the study will be presented using a CONSORT diagram and updated monthly throughout recruitment as a guide.

### Methods for additional analyses (e.g. subgroup analyses) {20b}

We will assess for any potential relationships between primary immune outcomes and secondary clinical outcomes.

### Methods in analysis to handle protocol non-adherence and any statistical methods to handle missing data {20c}

An investigation of missing data will be undertaken to understand study withdrawals or missing appointments and whether these could be related to baseline factors or AEs. Where data are incomplete, existing data will be used for all analyses. There will be no attempt to impute missing data.

### Plans to give access to the full protocol, participant-level data and statistical code {31c}

The full protocol is available from the corresponding author on reasonable request.

Until publication of the study results, access to the full dataset will be limited to the Trial Management Group (TMG). Requests for data sharing with bona fide study teams outside of Newcastle University or NuTH will be considered by a Data Access Committee, with representation from the sponsor and Chief Investigator (CI). Data transfer will be subject to completion of a Data Sharing Agreement between Newcastle University and the end users.

There is no relevant statistical code to access.

## Oversight and monitoring

### Composition of the coordinating centre and trial steering committee {5d}

Study conduct at site will be led by the PI and supported by GCP trained and appropriately delegated research clinicians and nurses.

Newcastle Clinical Trials Unit (NCTU) manages the trial on behalf of the sponsor and will provide day-to-day support for the site and training, site initiation activities and routine monitoring activities.

The TMG will be responsible for the day-to-day running of the study and will consist of the CI, PI and delegated researchers, members of NCTU, sponsor and, as required, other members of the study team. The TMG will monitor all aspects of the conduct and progress of the study. TMG meetings will occur approximately monthly.

The Trial Steering Committee (TSC) will provide overall independent oversight of the study and will oversee study conduct and progress. The TSC will consist of an independent chair, together with at least two other independent members, a Patient and Public Involvement (PPI) representative and the CI. The TSC will meet approximately 6-monthly throughout the study.

### Composition of the data monitoring committee, its role and reporting structure {21a}

The Data Monitoring Committee (DMC) will consist of at least three independent members including an Independent Chair, an Independent Statistician and an Independent Clinician and will meet approximately 6-monthly throughout the study. The DMC will make recommendations to the TSC as to whether there are any ethical or safety issues that may necessitate changes to the study.

### Adverse event reporting and harms {22}

Participants will be asked to report AEs at every study visit and encouraged to contact the study team for any concerns between visits via 24-h telephone numbers.

All AEs occurring from point of consent to end of the last study-related assessment will be recorded in the AE eCRF and the participant’s medical records. All serious AEs will also be reported to sponsor and to the MHRA if applicable.

All SARs occurring from administration of TolDC_CitPep_ to last study-related assessment will be reported to sponsor and recorded in the eCRF and medical records.

The assessment of expectedness will be performed by the CI on behalf of the sponsor against the approved Reference Safety Information for the study.

Any suspected unexpected serious ARs will be reported to the MHRA and Research Ethics Committee (REC) by the sponsor.

### Frequency and plans for auditing trial conduct {23}

A Site Delegation Log will detail the responsibilities of each member of site staff working on the study.

Quality control will be maintained through adherence to sponsor and NCTU SOPs, study protocol, GCP principles, research governance and clinical trial regulations.

Monitoring to ensure appropriate trial conduct and data collection will be carried out by NCTU according to a documented monitoring plan. Electronic data will be stored in secure, password-protected computers. NCTU staff will use a combination of central monitoring, off-site monitoring and on-site monitoring visits to ensure the study is conducted in accordance with GCP and the study protocol.

The study will permit audit by representatives of the sponsor or inspection by regulatory authorities as required.

### Plans for communicating important protocol amendments to relevant parties (e.g. trial participants, ethical committees) {25}

It is the responsibility of the sponsor to determine if an amendment is substantial or not and study procedures must not be changed without the mutual agreement of the CI, sponsor and the TMG.

Substantial amendments will be submitted to the REC and/or MHRA (as appropriate) and will not be implemented until such approval(s) is/are in place.

Non-substantial amendments will be submitted to the Health Research Authority and will not be implemented until authorisation is received.

Substantial amendments and those minor amendments which may impact site will be submitted to the relevant NHS Research & Development Department. Amendment documentation will be provided to sites by NCTU.

### Dissemination plans {31a}

A final report will be provided to the sponsor and REC within 1 year of the end of the study, defined as completion of all study related activities including completion of biomarker sample analysis for all participants.

Study results will be made publicly available on the ISRCTN trial registry within 1 year of the end of the study.

## Discussion

TolDC therapy is an emerging cellular therapy aimed at reversing the fundamental abnormality of immune dysregulation in autoimmune disease, but more knowledge of the TolDC product is needed.

TolDC_CitPep_ are autologous monocyte-derived tolerogenic dendritic cells loaded with citrullinated self-peptides, an investigational advanced therapy medicinal product. In the phase I AuToDeCRA study, autologous synovial fluid was used as the autoantigen for TolDC loading but carried limitations, including the need for participants to have a knee joint effusion and uncertainty as to which antigen-specific T cells were targeted. Extensive work over the last two decades has identified numerous candidate autoantigens in RA. In AuToDeCRA-2, TolDC will be loaded with synthetic peptides, meaning more sophisticated immune monitoring tools can be leveraged alongside the recruitment of participants with lower disease activity, who may benefit most from tolerance-inducing strategies.

One challenge for the study is choosing appropriate outcome measures. Clinical tolerance is difficult to measure in autoimmunity, as it may not correlate with clinical measures in the short-term [14]. At present, there is no universally accepted tolerance biomarker, but measures of autoantigen-specific immunity may provide the most important insights. Early phase studies of tolerogenic therapies have demonstrated changes to antigen- specific cellular immunity after treatment, including alteration of cytokine profiles, reduced proliferative responses following in vitro stimulation [5, 7], and changes in circulating frequencies of autoreactive T cells [20]. Global changes to T cell populations (e.g. regulatory and effector subsets) may also occur, and would be more practical as a ‘companion biomarker’ than measuring autoreactive T cells. Whilst the exact signature of tolerance induction remains uncertain, the ability of a candidate therapy to induce immunomodulation is a prerequisite for efficacy. In AuToDeCRA-2, multiple technologies will be used to monitor the immune state and, whilst changes previously observed by others will support the efficacy of TolDC_CitPep_, the wealth of data produced in the study will be interpreted with a view to identifying novel candidate biomarkers.

We anticipate that recruitment of participants with few or no symptoms may present another challenge due to the intensive monitoring, the use of an experimental advanced therapy medicinal product (ATMP) and the involvement of procedures including leukapheresis. Consequently, a robust and multi-faceted recruitment plan has been developed. Limitations include the small number of participants; however, this is felt to be a realistic recruitment goal and adequate to address the aims of the study.

In summary, AuToDeCRA-2 is an early phase experimental medicine study of an ATMP with the main objectives being to compare routes of administration and to demonstrate the ability of TolDC_CitPep_ to induce immunomodulation. Knowledge gathered from this study will help define biomarker outcomes and address existing scientific gaps necessary to inform the design of future TolDC studies for RA and other conditions where restoration of immune tolerance is desired.

## Trial status

Recruitment commenced on 15th January 2024 and is expected to complete by April 2025. The current protocol is version 5.0, dated 31 st July 2024.

## Data Availability

Until publication of the study results, access to the full dataset will be limited to the Trial Management Group.

## Abbreviations

ACPA: Anti-Citrullinated Peptide Antibodies
ACR: American College of Rheumatology
AE: Adverse event
APC: Antigen-presenting cells
AR: Adverse reaction
ARA: American Rheumatism Association
ATMP: Advanced Therapy Medicinal Product
AuToDeCRA: Autologous Tolerogenic Dendritic Cells for Rheumatoid Arthritis
CI: Chief Investigator
Cit: Citrullinated
CMV: Cytomegalovirus
CRP: C-reactive Protein
DAS: Disease Activity Score
DC: Dendritic cell
DMC: Data Monitoring Committee
eCRF: Electronic Case Report Form
EDTA: Ethylenediaminetetraacetic acid
ESR: Erythrocyte sedimentation rate
EULAR: European Alliance of Associations for Rheumatology
FBC: Full blood count
FSH: Follicle-stimulating hormone
GCP: Good Clinical Practice
GDPR: General Data Protection Regulation
GMP: Good Manufacturing Practice
HIV: Human immunodeficiency virus
HTLV: Human T cell lymphotrophic virus
HLA: Human leukocyte antigen
ICF: Informed consent form
INR: International normalised ratio
ISF: Investigator Site File
ISRCTN: International Standard Randomised Controlled Trials Number
LFT: Liver function test
MHRA: Medicines and Healthcare products Regulatory Agency
Mg: Magnesium
NCTU: Newcastle Clinical Trials Unit
NHS: National Health Service
NIHR: National Institute of Health and Care Research
NuTH: Newcastle upon Tyne Hospitals NHS Foundation Trust
PBMC: Peripheral Blood Mononuclear Cell
PI: Principal Investigator
PIS: Participant Information Sheet
RA: Rheumatoid arthritis
REC: Research Ethics Committee
RhF: Rheumatoid factor
SE: Shared Epitope
SOP: Standard Operating Procedure
SPIRIT: Standard Protocol Items: Recommendations for Interventional Trials
TMG: Trial Management Group
TolDC: Tolerogenic Dendritic Cells
TolDC_CitPep_: Tolerogenic Dendritic Cells loaded with citrullinated self-peptides
TSC: Trial Steering Committee
TNF: Tumour necrosis factor
U&E: Urea and electrolytes
VAS: Visual analogue scale

## References

[1] Bell GM, Anderson AE, Diboll J, Reece R, Eltherington O, Harry RA, et al. Autologous tolerogenic dendritic cells for rheumatoid and inflammatory arthritis. Ann Rheum Dis. 2017;76(1):227–34. 27117700 10.1136/annrheumdis-2015-208456 PMC5264217

[2] Stanway JA, Isaacs JD. Tolerance-inducing medicines in autoimmunity: rheumatology and beyond. Lancet Rheumatol. 2020;2(9):e565–75. 38273619 10.1016/S2665-9913(20)30100-4

[3] Giannoukakis N, Phillips B, Finegold D, Harnaha J, Trucco M. Phase I (safety) study of autologous tolerogenic dendritic cells in type 1 diabetic patients. Diabetes Care. 2011;34(9):2026–32. 21680720 10.2337/dc11-0472 PMC3161299

[4] Jauregui-Amezaga A, Cabezón R, Ramírez-Morros A, España C, Rimola J, Bru C, et al. Intraperitoneal Administration of Autologous Tolerogenic Dendritic Cells for Refractory Crohn’s Disease: A Phase I Study. J Crohns Colitis. 2015;9(12):1071–8. 26303633 10.1093/ecco-jcc/jjv144

[5] Nikolic T, Zwaginga JJ, Uitbeijerse BS, Woittiez NJ, de Koning EJ, Aanstoot HJ, et al. Safety and feasibility of intradermal injection with tolerogenic dendritic cells pulsed with proinsulin peptide-for type 1 diabetes. Lancet Diabetes Endocrinol. 2020;8(6):470–2. 32723484 10.1016/S2213-8587(20)30104-2

[6] Willekens B, Presas-Rodríguez S, Mansilla MJ, Derdelinckx J, Lee WP, Nijs G, et al. Tolerogenic dendritic cell-based treatment for multiple sclerosis (MS): a harmonised study protocol for two phase I clinical trials comparing intradermal and intranodal cell administration. BMJ Open. 2019;9(9):e030309. 31501122 10.1136/bmjopen-2019-030309 PMC6738722

[7] Zubizarreta I, Flórez-Grau G, Vila G, Cabezón R, España C, Andorra M, et al. Immune tolerance in multiple sclerosis and neuromyelitis optica with peptide-loaded tolerogenic dendritic cells in a phase 1b trial. Proc Natl Acad Sci U S A. 2019;116(17):8463–70. 30962374 10.1073/pnas.1820039116 PMC6486735

[8] Steinman RM, Hawiger D, Liu K, Bonifaz L, Bonnyay D, Mahnke K, et al. Dendritic Cell Function in Vivo during the Steady State: A Role in Peripheral Tolerance. Ann N Y Acad Sci. 2003;987(1):15–25. 12727620 10.1111/j.1749-6632.2003.tb06029.x

[9] Anderson AE, Sayers BL, Haniffa MA, Swan DJ, Diboll J, Wang XN, et al. Differential regulation of naïve and memory CD4+ T cells by alternatively activated dendritic cells. J Leukoc Biol. 2008;84(1):124–33. 18430785 10.1189/jlb.1107744 PMC2504714

[10] Anderson AE, Swan DJ, Sayers BL, Harry RA, Patterson AM, von Delwig A, et al. LPS activation is required for migratory activity and antigen presentation by tolerogenic dendritic cells. J Leukoc Biol. 2009;85(2):243–50. 18971286 10.1189/jlb.0608374 PMC2700018

[11] Harry RA, Anderson AE, Isaacs JD, Hilkens CM. Generation and characterisation of therapeutic tolerogenic dendritic cells for rheumatoid arthritis. Ann Rheum Dis. 2010;69(11):2042–50. 20551157 10.1136/ard.2009.126383 PMC3002758

[12] Anderson AE, Swan DJ, Wong OY, Buck M, Eltherington O, Harry RA, et al. Tolerogenic dendritic cells generated with dexamethasone and vitamin D3 regulate rheumatoid arthritis CD4(+) T cells partly via transforming growth factor-β1. Clin Exp Immunol. 2017;187(1):113–23. 27667787 10.1111/cei.12870 PMC5167049

[13] Benham H, Nel HJ, Law SC, Mehdi AM, Street S, Ramnoruth N, et al. Citrullinated peptide dendritic cell immunotherapy in HLA risk genotype–positive rheumatoid arthritis patients. Science Translational Medicine. 2015;7(290):290ra87-ra87. 10.1126/scitranslmed.aaa9301 26041704

[14] Morgan AW, Hale G, Rebello PR, Richards SJ, Gooi HC, Waldmann H, et al. A pilot study of combination anti-cytokine and anti-lymphocyte biological therapy in rheumatoid arthritis. QJM. 2008;101(4):299–306. 18287112 10.1093/qjmed/hcn006

[15] Ridolfi R, Riccobon A, Galassi R, Giorgetti G, Petrini M, Fiammenghi L, et al. Evaluation of in vivo labelled dendritic cell migration in cancer patients. J Transl Med. 2004;2(1):27. 15285807 10.1186/1479-5876-2-27 PMC509425

[16] Gerstner C, Dubnovitsky A, Sandin C, Kozhukh G, Uchtenhagen H, James EA, et al. Functional and Structural Characterization of a Novel HLA-DRB1*04:01-Restricted α-Enolase T Cell Epitope in Rheumatoid Arthritis. Front Immunol. 2016;7:494. 27895642 10.3389/fimmu.2016.00494 PMC5108039

[17] James EA, Rieck M, Pieper J, Gebe JA, Yue BB, Tatum M, et al. Citrulline-specific Th1 cells are increased in rheumatoid arthritis and their frequency is influenced by disease duration and therapy. Arthritis Rheumatol. 2014;66(7):1712–22. 24665079 10.1002/art.38637 PMC4248674

[18] Schwenzer A, Jiang X, Mikuls TR, Payne JB, Sayles HR, Quirke A-M, et al. Identification of an immunodominant peptide from citrullinated tenascin-C as a major target for autoantibodies in rheumatoid arthritis. Ann Rheum Dis. 2016;75(10):1876–83. 26659718 10.1136/annrheumdis-2015-208495 PMC5036245

[19] Snir O, Rieck M, Gebe JA, Yue BB, Rawlings CA, Nepom G, et al. Identification and functional characterization of T cells reactive to citrullinated vimentin in HLA–DRB1*0401–positive humanized mice and rheumatoid arthritis patients. Arthritis Rheum. 2011;63(10):2873–83. 21567378 10.1002/art.30445 PMC3174345

[20] Sonigra A, Nel HJ, Wehr P, Ramnoruth N, Patel S, van Schie KA, et al. Randomized phase I trial of antigen-specific tolerizing immunotherapy with peptide/calcitriol liposomes in ACPA+ rheumatoid arthritis. JCI Insight. 2022;7(20):e160964. 36278483 10.1172/jci.insight.160964 PMC9714780

